# Evaluation of the Utility of Thromboelastography in a Tertiary Trauma Care Centre

**DOI:** 10.1155/2014/849626

**Published:** 2014-02-12

**Authors:** Arulselvi Subramanian, Venencia Albert, Deepak Agrawal, Renu Saxena, Ravindra Mohan Pandey

**Affiliations:** ^1^Department of Laboratory Medicine, Jai Prakash Narayan Apex Trauma Centre, AIIMS, New Delhi 110029, India; ^2^Department of Neurosurgery, Jai Prakash Narayan Apex Trauma Centre, AIIMS, New Delhi 110029, India; ^3^Department of Haematology, AIIMS, New Delhi 110029, India; ^4^Department of Biostatistics, AIIMS, New Delhi 110029, India

## Abstract

*Background*. Thromboelastography (TEG) unlike conventional coagulation assays evaluates the dynamic interaction of clotting factors and platelets indicating an overall clot quality. Literature assessing the efficacy of TEG in identifying trauma associated bleeding is lacking. *Aim*. To compare TEG with conventional plasma based coagulation tests and assess whether TEG can serve as a screening test or replace the conventional routine test. *Materials*. Retrospective data was collected for 150 severe trauma patients. Patients with known evidence of severe comorbidities, which may influence the outcome, were excluded. Detailed evaluation of the patient's clinical and laboratory records was conducted. Diagnostic characteristics such as sensitivity, specificity, and accuracy were calculated. *Results*. Fifty-one patients were defined as coagulopathic by the conventional coagulation test, 30 by the laboratory established range for TEG indices and 105 by manufactures range. Specificity and sensitivity for the laboratory established range for TEG were 29.4% and 84.8%; for manufactures range sensitivity was 74.5%, specificity was 32.3%. *Conclusion*. We observed that conventional coagulation assays are the most sensitive tests for diagnosis of coagulopathy due to trauma. However in emergency trauma situations, where immediate corrective measures need to be taken, coagulation parameters and conventional coagulation tests may cause delay. TEG can give highly specific results depicting the underlying coagulopathy.

## 1. Introduction

High frequency of established coagulopathy (30%) in multiply injured trauma patients has been reported, which accounts for up to 40% of all trauma-related deaths [1].

The standard laboratory screening tests of coagulation are the prothrombin time (PT) and activated partial thromboplastin time (aPTT), which can be affected adversely by poor sampling technique; they vary in their complexity, turnaround time for clinical decision-making, and result reproducibility and have a need for special preparation or personnel [2].

Thromboelastography (TEG) is a noninvasive diagnostic assay to evaluate the viscoelastic properties during blood clot formation and clot lysis, yielding a graphical and numerical output relating to the cumulative effects of various plasma and cellular elements of all phases of the coagulation and fibrinolysis [3].

Numerous studies have reported the utilization of TEG as a monitoring device for hemostasis and transfusion management in various clinical settings, for example, cardiac surgery [4], liver transplantation [5], identification of patients with overt disseminated intravascular coagulation [6], hypercoagulability, and prediction thromboembolic events in surgical patients [7]. Studies claim that TEG is a point of care device for rapid diagnosis and differentiation of hypercoagulable and hyperfibrinolytic conditions [8].

TEG's ability to assess hemostasis in whole blood renders it to be ideal for rapidly identifying patients with trauma induced coagulation and transfusion guidance [9], as trauma patients present a spectrum of different coagulopathies that can be identified by TEG [10]. Animal studies have depicted that the conventional laboratory tests most clinicians use to evaluate clotting function in trauma patients are inadequate to document the significant changes, due to hypothermia and hemorrhage [11].

As values of viscoelastic haemostatic assays (VHA) parameters differ in different ethnic populations, there is a lack of standard protocol based on VHA parameters in Indian population to guide transfusion of blood component therapy in bleeding patients. The purpose of this study was to compare TEG with respect to the conventional coagulation tests and assess whether TEG can serve as a screening test or replace the conventional routine test for the identification of coagulopathy due to trauma; therefore we retrospectively studied hemostasis in critically injured trauma patients, admitted in level 1 trauma care center.

Firstly we established a reference range for TEG indices in north Indian healthy volunteers. Patients were segregated as hypercoagulable and hypercoagulable with respect to the laboratory established reference range, manufacturer given range, and TEG coagulation index. Based on the ability of the above TEG ranges to identify coagulation abnormalities in comparison to the conventional coagulation tests, we calculated the diagnostic accuracy of thromboelastography in trauma patients.

## 2. Subjects and Methods

This was a retrospective study conducted at the Department of Laboratory Medicine, of a Level 1 Trauma Care Center, over a period of six months (January–June 2013).

### 2.1. Study Subjects

Trauma patients with severe trauma (*n* = 150) admitted to our level 1 trauma care center were retrospectively considered for the study, if their TEG was analyzed on admission. Patients between the age group of 16 and 50 years were included in the study. Patients receiving anticoagulation therapy with warfarin or antiplatelet agents, patients with known underlying coagulopathy, and patients with clinical evidence of brain death at the time of admission, or evidence of other severe comorbidities, such as liver disease, diabetes mellitus, and known history of hypertension, which may influence the outcome, were excluded.

### 2.2. Sample Processing

The samples used for TEG analysis were drawn on arrival of the patient to the emergency department, prior to any fluid administration. Platelet count was done on Sysmex XE-2100 hematology analyzer (Sysmex, Kobe, Japan). Prothrombin time (PT) and activated partial thromboplastin time (aPTT) were estimated on a STA Compact automated analyzer (DiagnosticaStago, France).

### 2.3. Thromboelastography

Thromboelastometry was performed on TEM-A automated thromboelastometer (FramarBiomedica, Rome, Italy), using whole blood nonadditive (360 *μ*L). All analyses were performed with TEG disposable cups and pins as devised by the manufacturer and measurements were performed within 4 minutes of sampling. The TEG analyzer was stopped after 60 minutes from the time the test started. TEG evaluates the physical properties of the clot, via the torsion in a pin connected with a mechanical electrical transducer, suspended in cup. As the blood sample clots, the changes in rotation of the pin are converted into electrical signals that a computer uses to create graphical output.

Reference values successfully established in our laboratory for four TEM parameters: reaction (*r*-time) time (time from start to initial fibrin formation); *k*-time (clot kinetics, measuring time taken for a certain level of clot strength is reached); *α*-angle (clot kinetics of clot buildup and cross-linking); and maximum amplitude (MA) (absolute clot strength), for 95% of 200 healthy volunteers (100 male and 100 female), were *r*-time: 1.8–14.2 (min), *k*-time: 0.7–7.3 (min), *α*-angle: 27.3–72.3 (°), and MA: 32.1–87.9 (mm) (Table 1).

### 2.4. Clinical Variables

All patients underwent detailed clinical evaluation using the patient's record, and data was complied which included age, gender, date of admission, description of injury, Glasgow coma score, and injury severity score.

Coagulopathy was defined as >1.5 times control PT & aPTT values and/or INR > 1.6. Depending on the shape of the TEG tracing, the hemostatic condition of a patient was defined as hypocoagulable (if 2 or more parameters are observed: prolonged *r*-time, prolonged *k*-time, decreased *α*-angle and/or MA) or hypercoagulable (if 2 or more of the following parameters are observed: short *r*-time, short *k*-time, increased *α*-angle and/or MA). The TEG coagulation index (CI), derived from a linear equation that combines all the TEG variables, was also calculated [12] based on which the haemostatic condition of a patient was defined as hypercoagulable (CI > 3); hypocoagulable (CI < −3).

### 2.5. Statistical Analysis

All patients were categorized as normal and outliers based on the normal range for the (1) plasma based routine coagulation test, (2) our laboratory established reference range, and (3) manufacturer's reference range for TEG parameters.

True positive (TP), true negative (TN), false negative (FN), and false positive (FP) were defined with conventional coagulation tests as standard. Sensitivity, specificity, and accuracy were described in terms of TP, TN, FN, and FP.

*Sensitivity.* (The percentage of all patients with disease present who have a positive test) = TP/(TP + FN) = (Number of true positive assessment)/(Number of all positive assessments).

*Specificity*. (The percentage of all patients without disease who have a negative test) = TN/(TN + FP) = (Number of true negative assessment)/(Number of all negative assessments).

*Accuracy*. (The efficiency of a test is the percentage of the times that the test gives the correct answer compared to the total number of tests) = (TN + TP)/(TN + TP + FN + FP) = (Number of correct assessments)/(Number of all assessments).

The other two basic measures of diagnostic accuracy, that is, positive predictive value (PPV) and negative predictive value (NPV), which are related to sensitivity and specificity through disease prevalence, were also calculated. The predictive value of a test is a measure (%) of the times that the value (positive or negative) is the true value; that is, the percent of all positive tests that are true positives is PPV and NPV is the proportion of negative test results that are true negative:

 Positive Predictive Value = TP/(TP + FP)
 Negative Predictive Value = TN/(FN + TN).

## 3. Results

### 3.1. Patient Characteristics

One hundred and fifty injured patients presented to the emergency department from January through June 2013 were included in the study. Average age was 36.2 ± 15.4 years, 140 male and 10 female. 55.3% had incurred injury to the head (HI) of which 64 (77.1) had severe HI. Average injury severity score was observed to be 28.4 ± 11.3; mean Glasgow coma scale (GCS) was 7.85 ± 3.2Table 2.

### 3.2. Coagulation Parameters

PT, aPTT, and TEG parameters for all patients are shown in Table 2.

Fifty-one patients were defined as coagulopathic by the conventional coagulation test, with an average Hb of 10.2 ± 3.15 mm/Hg, platelet count 144.7 ± 87.6 lakh, PT 21.6 ± 5.4 sec, aPTT 38.8 ± 14.9 sec, and INR 1.8 ± 0.6.

Thirty patients were defined as coagulopathic by TEG based on their haemostatic conditions, out of which twelve were hypercoagulable and eighteen hypocoagulable. This was done on the basis of the reference range that was established in our laboratory for Indian population; however when we applied the reference range of the manufacturer, there were 105 patients who developed coagulopathy, out of which 53 were hypercoagulable and 52 were hypocoagulable.

Patients were also defined as coagulopathic based on the TEG coagulation index; we observed that 73 were hypercoagulable and 7 were hypocoagulable.

On comparing the identification of coagulopathy by conventional test to TEG (with laboratory reference range) we observed 15 patients to be true positive (TP), 15 were false positive (FP), whereas 84 were true negative (TN) and 36 were false negative (FN); based on the above defined formulas the sensitivity was observed to be 29.4%, specificity was 84.8%, PPV was observed to be 50%, and 70% was the NPV. The Efficiency of a Test Result was observed to be 66%.

When the identification of coagulopathy by conventional to manufacturers range for TEG indices was compared, we observed 38 patients to be true positive (TP), 67 were false positive (FP), whereas 32 were true negative (TN) and 13 were false negative (FN); based on the above defined formulas the sensitivity was observed to be 74.5%, specificity was 32.3%, PPV was observed to be 25.3%, and 71.1% was the NPV.

For TEG coagulation index we observed 23 TP, 57 FP, 42 TN, and 28 FN patients, with sensitivity 45.1% and specificity 42.4%, Table 3.

## 4. Discussion

Many tests provide information regarding coagulation status, including platelet count, prothrombin time, international normalized ratio, activated partial thromboplastin time, d-dimer, and fibrinogen levels. None is satisfactory in a trauma setting, as they take a considerable time to measure and represent a single point in a potentially ongoing process of bleeding. By the time the test results are available, the patient may already have entered an irreversible state of hypothermia, acidosis, and coagulopathy. As a result, there is growing interest in “point-of-care” testing, with capability for repeated measurements and rapid results within minutes [13].

PT and aPTT reflect the overall activity from plasma clotting factors involved in the extrinsic and intrinsic pathways, respectively. PT or aPTT do not adequately reflect coagulation defects from hypothermia and hemorrhage, as PT and aPTT are performed in platelet poor plasma, which exclude the interaction of platelets and other blood components, which significantly contribute to trauma coagulopathy [11].

TEG variables are interdependent, measuring the interaction of the coagulation cascade and platelets in whole blood rather than specific endpoints in centrifuged plasma samples. Enabling readily available analysis of coagulation in a shorter period of time than laboratory coagulation tests, thus providing useful and prompt identification of coagulation disorders [14].

Some studies report TEG to be a useful research tool in comparison to six common tests, hematocrit, platelet count, fibrinogen, PT, aPTT, and fibrin split degradation [14], suggesting a strong relationship. Similar results were found between TEG and standard laboratory tests of coagulation by Kang et al. [15], depicting a good correlation between *r*-time and aPTT [16].

However TEG continues to be a second-level hemostasis test due to the lack of its quality assurance procedures and that TEG is not validated, as far as international standards are concerned [17]. As a consequence of the dependence of TEG on manual procedures, its versatility in terms of the type of sample and different initiators that could be used resulting in difficulty to establish standards and reference values [18], it still persists as an expensive, nonvalidated point-of-care device [19]. There is also very limited published commercial data on performance with regard to assay precision or inter-assay variability that again leads to skepticism [20]. Other limitations associated with this technology are that, although it is sensitive to a deficiency or excess of coagulation factors, specific clotting factor deficiencies cannot be identified, have a relatively long measurement time, and require technical expertise [20].

The present study was conducted to evaluate the efficiency of thromboelastography in a trauma care setup to assess the changes in the hemostatic conditions of severely injured patients, in comparison to the routine coagulation tests.

Incidence of coagulopathy was observed to be 34% by the conventional coagulation tests; this decreased to 20% by our laboratory established range, indicating that routine assays recognize certain patients as coagulopathic which may not have the clinical presentation, and as TEG analyzes the whole hemostatic condition of the patient, it depicts these patients as normal; 70% developed coagulopathy according to the manufacturer's reference range, suggesting that many trauma patients would be incorrectly identified as coagulopathic by the manufacturer's reference values.

We found TEG to have a sensitivity of 29.41% and a specificity of 84.84% for trauma coagulopathy, using our reference range.

In their study to establish normal values for Canadian population, Scarpelini et al. reported a specificity of 81% for thromboelastography [18]. In a prospective study of 36 adult patients undergoing cardiopulmonary bypass (CPB) to determine the utility of TEG versus platelet studies and standard coagulation tests to more effectively discriminate patients likely to benefit from platelet or fresh frozen plasma (FFP) transfusion, Essell et al. [21] reported a sensitivity of (71.4%) and the specificity (89.3%) of TEG.

Sensitivity is the proportion of true positives that are correctly identified by a diagnostic test; it shows how good the test is at detecting a disease. Specificity is the proportion of the true negatives correctly identified by a diagnostic test, suggesting how good the test is at identifying normal (negative) condition. We observed that the range given by the manufactures has a higher sensitivity (74.5%) but a low specificity (32.3%) compared to our laboratory range, which indicates that the manufacturers range may identify coagulation abnormalities in a number of patients, because a test with high sensitivity tends to capture all possible positive conditions without missing anyone. However a low specificity suggests that these patients may not have clinical presentation of coagulopathy. Also high sensitivity but low specificity may result in that many patients who do not have coagulation abnormalities being identified as coagulopathic are then subjected to further investigation, using laboratory established range.

The major limitation of both sensitivity and specificity is that they are of no practical use when it comes to helping the clinician estimate the probability of disease in individual patients. Positive and negative predictive values describe a patient's probability of having a condition once the results of the tests for this condition are known [22]. In our present study TEG using the laboratory range had PPV of 50% and NPV of 70%; for the manufacturers range PPV of 25.3% and NPV of 71.1% were observed. The higher the PPV, the more likely a positive result is able to predict the presence of coagulopathy.

Both PPV and NPV vary with changing prevalence of disease. It will therefore be wrong for clinicians to directly apply published predictive values of a test to their own populations, when the prevalence of disease in their population is different from the prevalence of disease in the population in which the published study was carried out [22].

Accuracy measures how correct a diagnostic test identifies and excludes a given condition and can be determined from sensitivity and specificity with the presence of prevalence; it measures the degree of veracity of a diagnostic test on a condition [23]. We report an accuracy of 66% for TEG using laboratory established range and 46.6% for the manufacturers.

Limitations of the present study are that TEG investigations were not done in duplicate, as is the case for classical coagulation assays; we did not take into account the transfusion requirements; thus we could not assess the transfusion guidance using out TEG reference values.

## 5. Conclusions

The efficacy of classical coagulation test has been well established. However, point of care testing for hemostasis is emerging and its role in patient management remains to be demonstrated in prospective studies.

Based on the current study, it is concluded that for specific diagnosis of underlying etiology of bleeding, conventional coagulation tests remain the most sensitive tests compared to thromboelastography; however in emergency trauma situations, where immediate corrective measures need to be taken based on coagulation parameters and conventional coagulation tests may cause delay, TEG can give highly specific results depicting the underlying coagulopathy. However it is suggested that the reference range of TEG tests should be established for each lab and its results eventually should be confirmed by specific coagulation tests.

## Figures and Tables

**Table 1:** The derived reference range of thromboelastogram parameters and the reference range of thromboelastogram parameters predefined by the manufacturers.

Parameters	Predefined reference range by the manufacturers	Derived reference range volunteers
*r*-time (min)	5–11	1.8–14.2
*k*-time (min)	1.5–4.5	0.7–7.3
*α* angle (°)	44.5–66	27.3–72.3
Maximum amplitude (mm)	53–70	32.1–87.9

**Table 2:** Baseline characteristics.

Study parameters	Mean ± S.D. (*n* = 150)
Age	36.2 ± 15.4
Gender*	
Male	140 (93.3)
Female	10 (6.6)
Type of injury*	
Head	83 (55.3)
Trauma	17 (11.3)
Extremities	20 (13.3)
Polytrauma	30 (20.0)
Glasgow coma scale (*n* = 32)	7.8 ± 3.20
Severity of head injury* (*n* = 32)	
Severe	64 (77.1)
Moderate	14 (16.8)
Mild	5 (6.0)
New injury severity score	28.4 ± 11.3
Hemoglobin (g/dL)	11.3 ± 2.6
Platelet (lkh)	166.4 ± 82.2
Prothrombin time (sec)	17.5 ± 4.4
Activated partial thromboplastin time (sec)	31.0 ± 10.8
INR	1.3 ± 0.4
Coagulopathy*	
Yes	51 (34.0)
No	99 (66.0)
*r*-time (sec)^#^	5.7 (0.7–23)
*k*-time (sec)^#^	3 (0–52.3)
*α* angle (°)	48.8 ± 19.5
Maximum amplitude (mm)	56.5 ± 27.4

*Data expresses as frequency (percentage); ^#^as median (IQR).

**Table 3:** Sensitivity, specificity, and accuracy of thromboelastography in comparison to the convention coagulation assay (PT/aPTT).

Characteristics	Laboratory reference range % (95% CI)	Manufacturers range % (95% CI)	TEG using coagulation index % (95% CI)
Sensitivity (%) TP/(TP + FN)	29.4 (18.7–43)	74.5 (61.1–84.4)	45.0 (32.2–58.6)
Specificity (%) TN/(TN + FP)	84.8 (76.5–90.6)	32.3 (23.9–42.0)	42.4 (33.1–52.2)
Positive predictive value (%) TP/(TP + FP)	50.0 (33.1–66.8)	25.3 (27.6–45.7)	28.7 (19.9–39.4)
Negative predictive value (%) TN/(FN + TN)	70.0 (61.2–77.4)	71.1 (56.6–82.2)	60.0 (48.2–70.6)
Diagnostic accuracy (%) (TN + TP)/(TN + TP + FN + FP)	66.0 (58.1–73.1)	46.6 (38.8–54.6)	43.3 (35.6–51.3)
